# Nanoelectropulse-driven membrane perturbation and small molecule permeabilization

**DOI:** 10.1186/1471-2121-7-37

**Published:** 2006-10-19

**Authors:** P Thomas Vernier, Yinghua Sun, Martin A Gundersen

**Affiliations:** 1 Department of Electrical Engineering-Electrophysics, Viterbi School of Engineering, University of Southern California, Los Angeles CA, 90089-0271, USA; 2 MOSIS, Information Sciences Institute, Viterbi School of Engineering, University of Southern California, Los Angeles, CA 90292-6695, USA; 3 Department of Materials Science and Engineering, Viterbi School of Engineering, University of Southern California, Los Angeles CA, 90089-0271, USA

## Abstract

**Background:**

Nanosecond, megavolt-per-meter pulsed electric fields scramble membrane phospholipids, release intracellular calcium, and induce apoptosis. Flow cytometric and fluorescence microscopy evidence has associated phospholipid rearrangement directly with nanoelectropulse exposure and supports the hypothesis that the potential that develops across the lipid bilayer during an electric pulse drives phosphatidylserine (PS) externalization.

**Results:**

In this work we extend observations of cells exposed to electric pulses with 30 ns and 7 ns durations to still narrower pulse widths, and we find that even 3 ns pulses are sufficient to produce responses similar to those reported previously. We show here that in contrast to unipolar pulses, which perturb membrane phospholipid order, tracked with FM1-43 fluorescence, only at the anode side of the cell, bipolar pulses redistribute phospholipids at both the anode and cathode poles, consistent with migration of the anionic PS head group in the transmembrane field. In addition, we demonstrate that, as predicted by the membrane charging hypothesis, a train of shorter pulses requires higher fields to produce phospholipid scrambling comparable to that produced by a time-equivalent train of longer pulses (for a given applied field, 30, 4 ns pulses produce a weaker response than 4, 30 ns pulses). Finally, we show that influx of YO-PRO-1, a fluorescent dye used to detect early apoptosis and activation of the purinergic P2X_7 _receptor channels, is observed after exposure of Jurkat T lymphoblasts to sufficiently large numbers of pulses, suggesting that membrane poration occurs even with nanosecond pulses when the electric field is high enough. Propidium iodide entry, a traditional indicator of electroporation, occurs with even higher pulse counts.

**Conclusion:**

Megavolt-per-meter electric pulses as short as 3 ns alter the structure of the plasma membrane and permeabilize the cell to small molecules. The dose responses of cells to unipolar and bipolar pulses ranging from 3 ns to 30 ns duration support the hypothesis that a field-driven charging of the membrane dielectric causes the formation of pores on a nanosecond time scale, and that the anionic phospholipid PS migrates electrophoretically along the wall of these pores to the external face of the membrane.

## Background

Nanosecond, megavolt-per-meter pulsed electric fields nondestructively perturb the intracellular environment, causing calcium bursts [1,2], eosinophil sparklers [3], vacuole permeabilization [4], and the appearance of apoptotic indicators such as release of cytochrome c into the cytoplasm [5] and caspase activation [6,7]. In addition to these effects in the cell interior, nanoelectropulse exposure also induces phosphatidylserine (PS) externalization – translocation of PS from the cytoplasmic face of the plasma membrane to the cell exterior – a normal event in platelet activation and blood coagulation [8], a diagnostic feature of apoptotic cells which serves as a physiological semaphore for their phagocytic removal [9], and a means of intramembrane signal transduction in lymphocytes [10].

Previously we have demonstrated, using annexin V binding as a measure of PS externalization [11], that exposing Jurkat T cells to 50, 7 ns pulses causes immediate PS translocation with field strengths above 2 MV/m, with little response to 1 MV/m pulses [12]. Increasing the pulse count produces a proportionally greater response [7]. The presence of 5 millimolar EGTA in the cell suspension during pulse exposure does not reduce subsequent annexin V binding, indicating that nanoelectropulse-induced PS translocation does not require calcium in the external medium [12]. In addition, by employing the fluorescent dye FM1-43 as an indicator of relative changes in the phospholipid composition of the external leaflet of the plasma membrane [13], we have achieved real-time microscopic visualization of pulse-induced phospholipid scrambling, which occurs within milliseconds of pulse delivery, is pulse count- and field-dependent, and is confined always to the anode-directed pole of the cell [14].

PS is normally confined in healthy cells to the inner leaflet of the membrane lipid bilayer. This asymmetry is enforced by an energy barrier of approximately 100 kJ/mol [15] that impedes transport of the charged phospholipid head group across the hydrophobic interior of the membrane [16,17]. Physiological regulation of PS distribution in the membrane is maintained by enzymatic activities that both scramble and restore the normal asymmetry [18-21], and is associated with the release of Ca^2+ ^from internal stores [22], the proximity of depolarized mitochondria [23], and the restructuring of membrane-cytoskeletal attachments [24,25].

Electroporation of cells under conditions conducive to the stabilization of large pores, using relatively low-field pulses of microsecond or millisecond duration, causes PS externalization in erythrocyte membranes [26,27]. At the site of a long-lasting (milliseconds to seconds) pore, the inner and outer membrane leaflets align and merge, and PS can rapidly diffuse [28,29] along this now contiguous surface.

What about nanosecond pulses? For a given pulsed bioelectrical experimental system at the cellular level, if the pulse duration is short enough relative to the charging time constant of the resistive-capacitive network formed by the conductive intracellular and extracellular fluids and the cell membrane dielectric, then the electric field is expressed across the intracellular space, while at the same time the potential difference that develops across the external membrane, initially very small, may not reach porating voltage levels [30,31]. For human lymphocytes in growth medium the charging time constant for the plasma membrane lies in the range of 50 to 100 ns, using a dielectric shell model of the cell [32,33]. Thus it is not surprising that previous reports have shown that nanosecond, megavolt-per-meter pulses (7–300 ns, 2.5–30 MV/m) also induce PS translocation, but *without *significant poration of the external membrane [5,7], although a mechanism for this phospholipid rearrangement after submicrosecond pulse exposure remains to be established.

**Figure 1:**
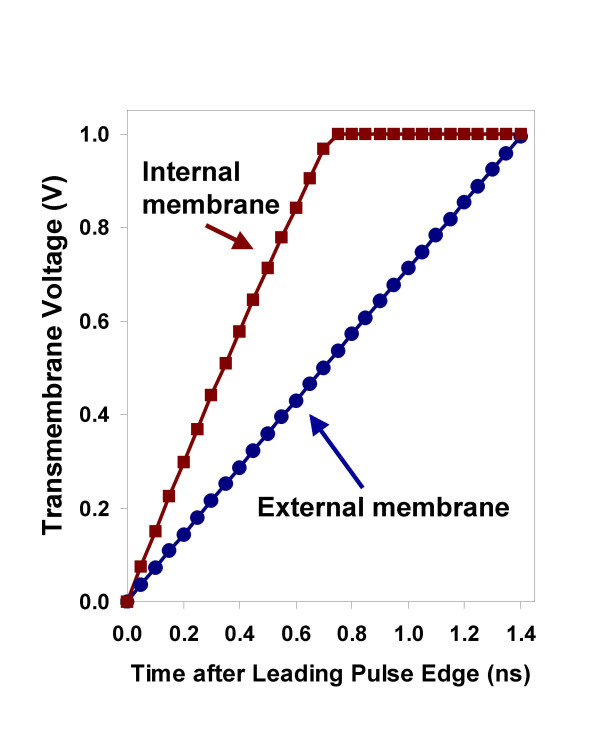
**Pulse-driven transmembrane potential**. Voltages across the external membrane of a human lymphocyte with a radius of 5.0 μm and an intracellular membrane-bound structure with a radius of 0.5 μm, after a pulsed field step from 0 to 8 MV/m in growth medium, calculated from a simple dielectric shell model of the cell [7,12], showing that the potential across the external membrane can be expected to reach 1 V in less than 2 ns. Electrical parameters are derived from values in [33]. The membranes of intracellular vesicles or organelles like mitochondria will charge more quickly because the capacitance of these smaller structures is less than that of the cell membrane.

We can estimate, however, that a pulsed field with amplitude on the order of a few megavolts per meter, which is required in order to produce significant intracellular fields and perturbative potentials across organelle membranes [7], will charge the plasma membrane of a human lymphocyte in nutrient medium to the critical, porating transmembrane potential of approximately 1 V [34,35] in less than 2 ns (Fig. 1). The values plotted in Fig. 1 are derived from the simple model equation

V_m_(t) = 1.5 E_0 _a cos θ [1 - exp(-t/τ_m_)],     (1)

where *E*_0 _is the applied electric field, *a *is the cell radius, *θ *is the position angle relative to the electric field, *t *is the time after the initiation of the pulse, *τ*_m _is the membrane charging time constant, and values for the parameters reflect the electrophysical characteristics of lymphocytes in serum (a = 5.0 μm, θ = 0, τ_m _= 83 ns). As we stressed previously [12], the cell charging time constant is not precisely known, the dielectric shell model embodies only the most general electrophysical properties of the system, and a real population of cells is heterogeneous in size, shape, and physiological state. Nevertheless we may draw from these numbers, in agreement with those obtained from more sophisticated models [36,37], the expectation that even a pulse of nanosecond duration will charge the external membrane of the cell to supraphysiological, porating potentials when the pulsed field strength is on the order of megavolts per meter. The conclusion follows that high-field pulses in the picosecond regime will be required in order to avoid producing significant (porating) plasma membrane potentials with megavolt-per-meter pulses.

Until sub-nanosecond pulse generators that can develop the requisite potentials across biological loads are available, we cannot make the simplifying assumption that the external membrane is unaffected by ultra-short, high-field electric pulses. In fact, all of the evidence we have assembled here and elsewhere points to the pulse-induced increase in transmembrane voltage as the primary driver for the PS translocation observed immediately after nanoelectropulse exposure in lymphocyte-derived cell lines.

In this paper we describe the membrane restructuring responses of cells to 3 ns and 4 ns pulses with applied field amplitudes up to 8 MV/m. Even with these very short pulses we must expect that the potential across the plasma membrane exceeds levels associated with increases in membrane conductance (poration) [38]. Although it has not been clearly established whether poration is occurring under these circumstances or whether poration enables or facilitates nanoelectropulse-induced phospholipid rearrangement and PS externalization, our results here are consistent with nanosecond pulse-induced permeabilization of the cell membrane to small molecules. This new evidence suggests that some poration, undetectable with conventional methods, is occurring even with pulse durations as short as 3 ns, if the electric field is high enough, and that these nanoelectropulse-induced membrane defects may be the sites for the migration of PS to the outer leaflet of the lipid bilayer.

## Results

We tested four overlapping hypotheses relating to the mechanism of nanoelectropulse-induced phospholipid rearrangement and PS externalization: 1. that the PS molecule is driven across the membrane lipid bilayer by the action of the electric field on the negatively charged PS head group, either directly across the hydrophobic interior of the membrane or as an electrophoretic migration at transient membrane nanopores; 2. that nanosecond pulse-induced membrane restructuring and PS translocation depends on the charging of the membrane dielectric; 3. that pulse-driven PS externalization does not require intracellular or extracellular calcium even with pulse durations as short as 3 ns; 4. that the formation of transient, nanometer-scale pores facilitates PS translocation, either through electrophoresis in the pore walls or as a result of the phospholipid scrambling that accompanies pore formation and subsequent passive "lateral" diffusion from the PS-rich inner leaflet along the solvent-facing surface of the pore to the outer leaflet of the membrane.

### Pulse polarity and membrane perturbation patterns are consistent with electric field-driven PS translocation

**Figure 2:**
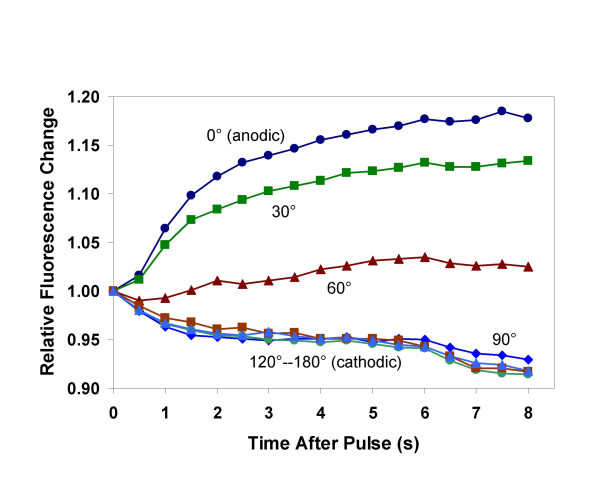
**Circumferential variation of membrane perturbation and PS externalization after pulse exposure**. Pulse-induced changes in FM1-43 fluorescence intensity after delivery of 4, 30 ns, 2.5 MV/m pulses were extracted photometrically from microscope images by defining tangential regions of interest approximately 2 μm by 0.5 μm at 0°, 30°, 60°, 90°, 120°, 150°, and 180° around the circumference of a Jurkat T cell image, with 0° at the anode pole. This data was extracted from the results summarized in Fig. 4 of [14] to demonstrate for a single cell both the kinetics of the FM1-43 fluorescence intensification after pulse exposure, and the angular dependence of the response.

**Figure 3:**
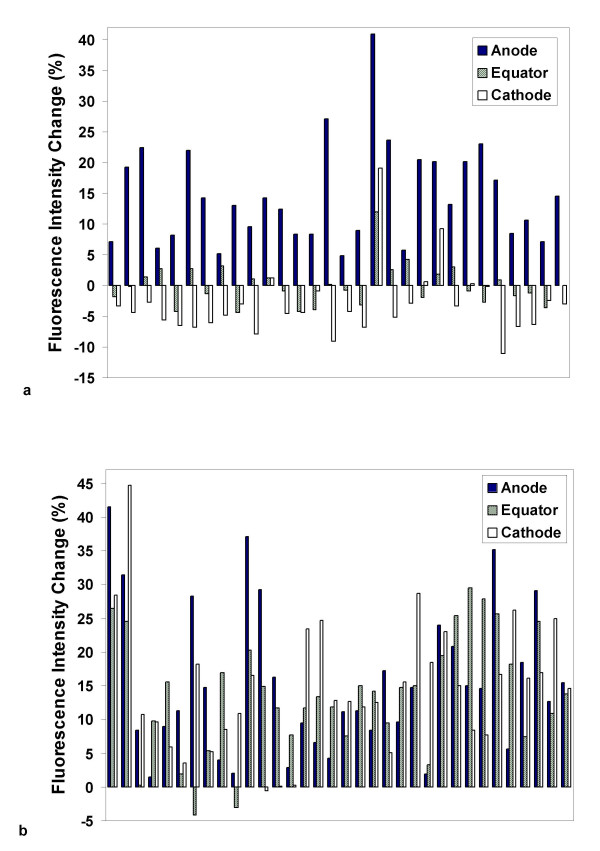
**Anodic polarity of phospholipid rearrangement and PS externalization**. Anodic, equatorial, and cathodic PS externalization responses for individual Jurkat cells exposed to 50, 3 ns, 6 MV/m pulses were extracted photometrically from microscope images by defining tangential regions of interest approximately 2 μm by 0.5 μm at 0°, 90°, and 180° around the circumference of a Jurkat T cell, with 0° at the anode pole. Results are shown for images captured at 7 s, before externalized PS has diffused laterally away from the translocation sites (a), and at 5 min, showing the overall increased FM1-43 fluorescence after the translocated PS has become distributed evenly around the cell (b). Each group of three bars (anode, equator, cathode) represents the data from a single cell [29 cells for (a); 33 cells for (b)]. The rightmost group of three bars in each graph represents the mean of all cells in the data sets. A similar pattern is observed with 4 ns pulses.

We have previously reported that PS externalization after exposure to unipolar, 30 ns, 2.5 MV/m pulses is confined to the anode-facing pole of the cell [14]. The immediate post-pulse time course of this anodic polarization for a 30 ns pulse is shown in Fig. 2. We now report that 3 and 4 ns pulses produce a similar pattern. For a few seconds following pulse exposure, FM1-43 fluorescence intensification, which we interpret to indicate phospholipid rearrangement and PS externalization, is apparent at the anode pole only and is never observed at the cathode pole. Within a minute the increase in FM1-43 fluorescence is distributed uniformly in the cell membrane (Fig. 3).

Although we observe a similar anodic polarization in images of cells pulsed in the presence of fluorescent-tagged annexin V [39], the high fluorescence background and the slower kinetics of annexin V binding lead us to prefer membrane-bound FM1-43 fluorescence as a rapidly responding marker for perturbation of the lipid bilayer. FM1-43, which partitions between the aqueous medium and the cytoplasmic membrane has been identified as an effective and rapidly responsive indicator of PS translocation in Jurkat cells [16]. Translocation of the negatively charged PS head group to the outer face of the membrane provides additional electrostatic attraction for the quaternary ammonium of FM1-43, resulting in more dye binding per unit area and a concomitant increase in fluorescence emission. The fluorescence quantum yield of FM1-43 increases by several orders of magnitude on membrane binding [40], facilitating real-time microscopic observations of changes in externalized PS by eliminating the need for washing. Because the increased FM1-43 fluorescence associated with PS externalization results from the insertion of additional dye molecules into the membrane, the time resolution for observations of PS translocation is limited by the rate of diffusion of FM1-43 to the medium-membrane interface and by the rate of insertion of the FM1-43 tail into the array of phospholipids at the membrane surface. Although FM1-43 does not have the PS specificity of annexin V, observations of membrane restructuring at time scales of seconds or less than a second are greatly facilitated by the more rapid kinetics of the FM1-43 response [41]. Extensions of this study will be necessary to define more precisely the details of electric pulse-driven membrane reorganization, but all of our evidence is consistent with the interpretation that PS externalization is a significant component of that reorganization.

**Figure 4:**
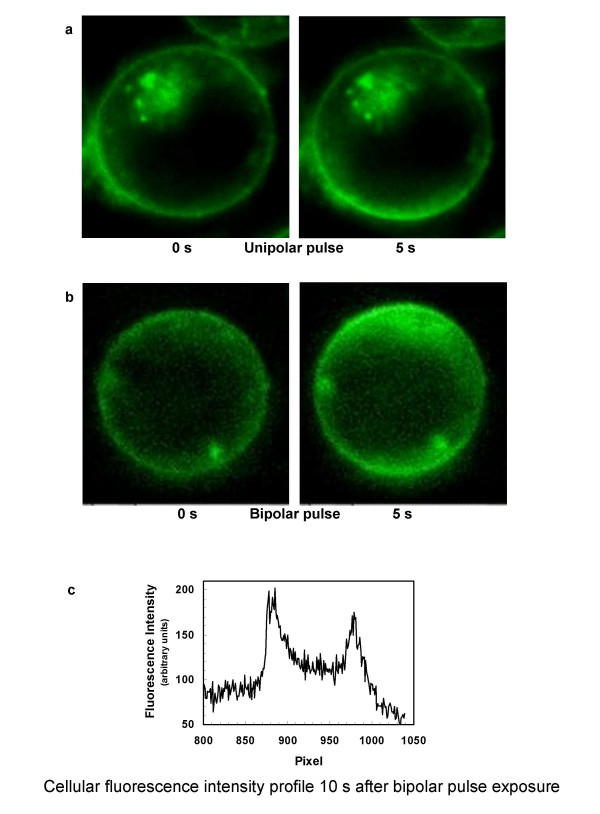
**Membrane phospholipid perturbation patterns in Jurkat T lymphoblasts after unipolar and bipolar pulse exposure**. (a) FM1-43 fluorescence increase appears only at the anode (bottom) pole of the cell after 4, 30 ns, 2.5 MV/m pulses. (b) Bipolar pulses (50, 15 ns, 2.8 MV/m) produce phospholipid rearrangement and PS externalization at both electrode-facing poles of the cell. (c) Cellular fluorescence intensity profile 10 s after bipolar pulse exposure. The frequency of pulse delivery was 10 Hz. Each bipolar pulse was delivered as a pair of pulses, first negative, then positive, each 15 ns wide. Total energy delivered for the unipolar and bipolar pulses was approximately the same, and no poration is observed by conventional methods at these pulse exposure levels.

To confirm the dependence of lipid bilayer perturbation and associated PS externalization on the polarity of the pulsed electric field, we monitored FM1-43 fluorescence intensification patterns in Jurkat T cells during delivery of pulses of alternating polarity. In contrast to the effect of unipolar pulses, a bipolar pulse train disturbs the phospholipid order at both electrode-facing poles of the cell (Fig. 4), consistent with an essential vectorial component in the observed perturbations of anionic phospholipids in the cell membrane by externally applied pulsed electric fields. These results are consistent with the first hypothesis above, which posits a direct interaction between the pulsed electric field and lipid bilayer components rather than a secondary phenomenon triggered by and developing subsequent to the pulse exposure, extending previous observations to higher fields and shorter pulses, and directly demonstrating the effect of reversing the polarity of the field.

### Pulse width and pulse amplitude. Shorter pulses require higher fields, consistent with a time-dependent charging of the membrane dielectric

**Figure 5:**
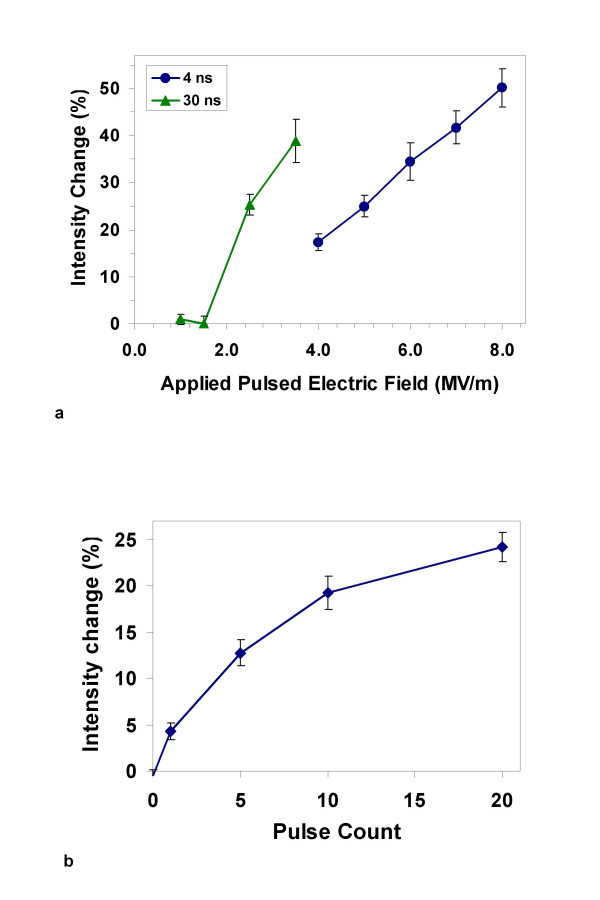
**Phospholipid bilayer re-ordering and PS translocation with shorter pulses requires higher amplitudes**. (a) Relative, integrated, whole-cell, FM1-43 fluorescence intensities 5 min after pulse exposure (4, 30 ns pulses; 30, 4 ns pulses; repetition rate = 10 Hz) indicate that to produce an equivalent amount of lipid bilayer perturbation and PS externalization, shorter pulses (4 ns) must have higher fields than longer pulses (30 ns). Note that the total pulse on time for the two pulse widths was adjusted to be approximately the same – 120 ns (4 × 30 ns = 30 × 4 ns). The architecture of the pulse generators does not permit increasing the amplitude of the 30 ns pulses above 3.5 MV/m or decreasing the amplitude of the 4 ns pulses below 4 MV/m. (b) Pulse count dependence of FM1-43 fluorescence intensification 5 minutes after exposure to 3 ns, 6.0 MV/m pulses, repetition rate = 10 Hz.

In previous studies we noted a low-field threshold for pulse-induced PS externalization, roughly corresponding to the critical transmembrane potential for electroporation [12,14], and we have suggested that charging of the membrane to this critical voltage, with or without the associated formation of electropores, may be essential for nanosecond pulse-driven PS translocation. We have tested this idea by comparing the field dependence of pulse-induced phospholipid rearrangement and PS externalization in Jurkat cells for 4 ns and 30 ns pulses. To produce the same peak transmembrane potential for cells with a given charging time constant, a 4 ns pulse must have a larger amplitude than a 30 ns pulse. Although many factors beyond the simple charging of the membrane dielectric may contribute to the observed rearrangement of membrane constituents indicated by FM1-43 fluorescence intensification, the data (Fig. 5a) clearly demonstrate that for time-equivalent exposures (4, 30 ns pulses and 30, 4 ns pulses) a considerably higher field is required to produce a measurable response when the pulse width is reduced, consistent with a correlation between pulse-induced membrane potential and the extent of phospholipid rearrangement and PS translocation. This time- and field-dependence is consistent with the second, dielectric charging hypothesis above. The cumulative effect of multiple pulse exposures, reported previously for 20 and 30 ns pulses [7,14] is reflected in FM1-43 fluorescence intensification also with 3 ns pulses (Fig. 5b).

### Nanoelectropulse-induced membrane restructuring and PS externalization does not require extracellular or intracellular calcium

**Figure 6:**
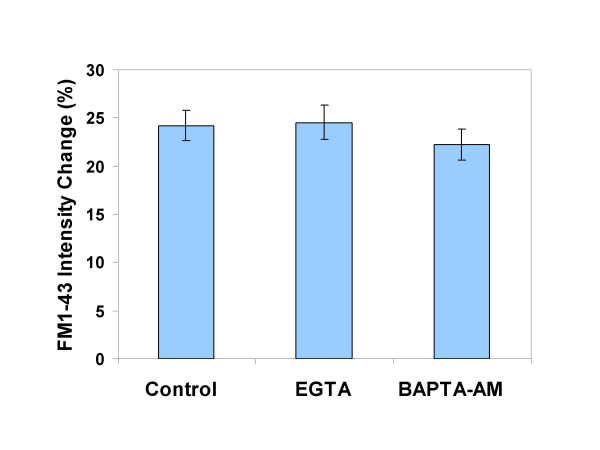
**Nanoelectropulse-induced phospholipid rearrangement and PS externalization does not require calcium**. FM1-43 fluorescence intensification was photometrically measured 5 min after exposure to 20, 3 ns, 6.0 MV/m pulses. Experimental samples had either 5 mM EGTA in the medium (added immediately before pulse delivery) or were loaded with 10 μM BAPTA-AM for 60 min before pulsing.

We have previously shown that PS externalization occurs after exposure to 30 ns pulses with amplitudes greater than 1 MV/m even in the presence of extra- and intracellular calcium chelating agents [14]. Here we present evidence for a similar calcium independence of phospholipid re-ordering with much shorter, 3 ns, 6 MV/m pulses. Neither EGTA at 5 mM in the culture medium nor BAPTA, loaded into the cytoplasm as the acetoxymethyl ester at 10 μM, inhibited the integrated, whole-cell, FM1-43 fluorescence intensification measured 5 min after a 20-pulse exposure (Fig. 6).

### Small-molecule electropermeabilization after nanosecond pulse exposure

The failure of previous efforts to demonstrate influx of ions and small molecules from the medium into the cytoplasm after nanosecond, megavolt-per-meter pulse treatment [1,7,12,14] has not conclusively established that nanoporation – the generation of nanometer-diameter pores with lifetimes of nanoseconds – does not occur, but rather only that it has not been detectable under the conditions of our experiments. Because even relatively small amounts of calcium entering or sodium leaving the cytoplasm can trigger a physiologically large response through cascading signal transduction chains or membrane depolarization, we have continued to seek evidence that nanosecond pulsed electric fields generate pores that might conduct ions or larger species into (or out of) the cell even for a few nanoseconds.

**Figure 7:**
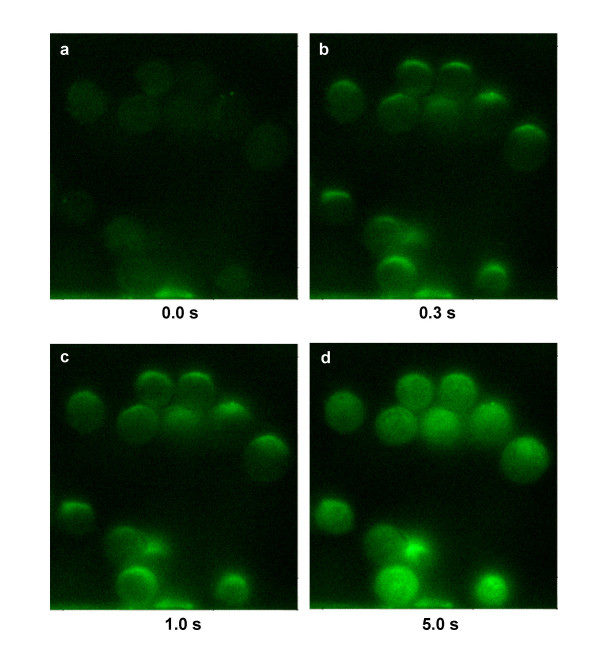
**High-field, nanosecond pulses at high repetition rates permeabilize cell membranes, permitting entry of the small molecule YO-PRO-1 into the cell**. Fluorescence microscopic images of Jurkat cells in growth medium containing YO-PRO-1 (5.0 μM) at 0.0, 0.3, 1.0, and 5.0 s after exposure of the cells to 100, 4 ns, 8 MV/m pulses at a repetition rate of 1 kHz. Influx of YO-PRO-1 occurs primarily at the anode pole of the cells.

**Figure 8:**
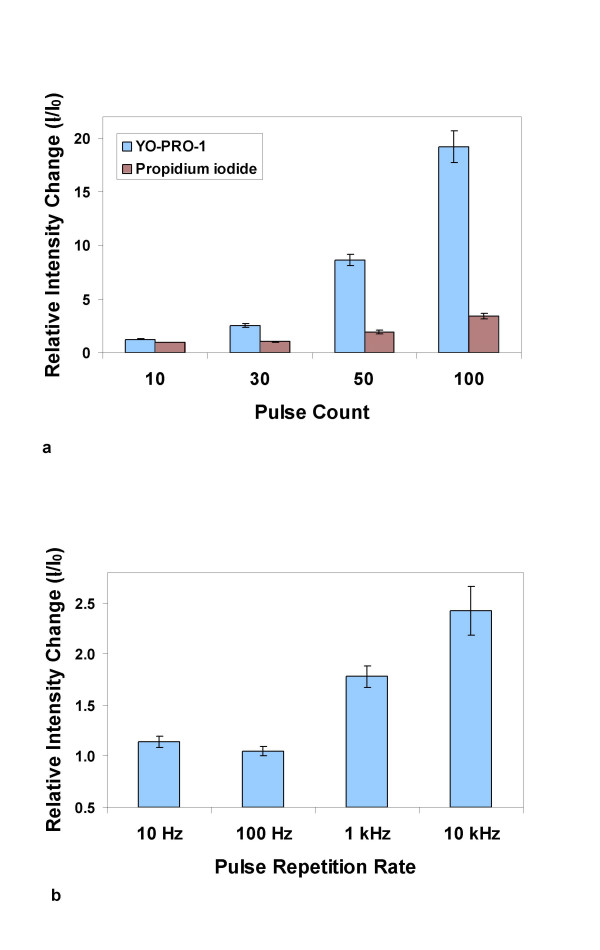
**Small molecule permeabilization by multiple, high-field, nanosecond pulses**. Fluorescence microscopic images of individual Jurkat cells in growth medium containing YO-PRO-1 (5.0 μM) or propidium iodide (7.5 μM) were captured immediately before and 5 min after exposure of the cells to 4 ns, 8 MV/m pulses and the fluorescence intensity change for each cell was measured by photometric integration. (a) Measurable influx of YO-PRO-1 occurs after 30 pulses delivered at 1 ms intervals (1 kHz repetition rate). At higher pulse counts, permeability of the cells to YO-PRO-1 increases and propidium iodide influx can be detected. (b) YO-PRO-1 influx is not detected when 30 pulses are delivered at 10 Hz and 100 Hz.

We now report the influx of YO-PRO-1, a fluorescent dye that binds to nucleic acids, and which is also a sensitive indicator of early apoptosis [42] and of activation of P2X_7 _(purinergic) receptor channels [43], after exposure of Jurkat cells to a sufficiently large number (30) of 4 ns pulses at fields above 6 MV/m at high pulse repetition rates (1 kHz), under conditions where detectable levels of propidium iodide continue to be excluded from the cell interior (Fig. 7). Propidium iodide entry is also observed (Fig. 8) with even higher nanoelectropulse counts (100) at the highest fields and repetition rates used in these studies (8 MV/m, 10 kHz). This distinction between YO-PRO-1 and propidium iodide, which are comparable in size and molecular weight and are both divalent cations in aqueous solution at neutral pH, suggests that nanoelectropulse-induced permeabilization may involve more than simple poration of the membrane.

To test the possibility that nanosecond pulsed electric fields in these experiments are activating the P2X_7 _receptors, allowing YO-PRO-1 to cross the membrane through these channels, we pulsed cells in the presence of the P2X_7 _receptor inhibitor Brilliant Blue G [44]. No measurable effect on YO-PRO-1 influx was observed even at Brilliant Blue G concentrations as high as 100 μM.

This new evidence for membrane permeabilization after nanosecond electric pulse exposure (nanoporation) is consistent with the pore-facilitated phospholipid translocation proposed in the fourth hypothesis above.

## Discussion

The idea that very short electric pulses can bypass the capacitance of the plasma membrane and reach the cell interior has stimulated an expanding variety of experimental studies and the development of a new technology [45]. Calculations based on a simple dielectric shell model of the cell indicate, however, that even for pulse durations as short as 2 ns, the megavolt-per-meter external fields needed to develop significant potentials across intracellular structures will charge the plasma membrane to the critical voltage associated with the appearance of high conductance pores after longer pulse exposures (Fig. 1). It is reasonable to expect then that nanoelectropulse manipulations of cells will have effects both in the cytoplasm and at the external cell membrane.

While most studies of nanosecond, megavolt-per-meter bioelectrical phenomena have focused on intracellular events (apoptosis, calcium signaling, intracellular membrane permeation, nuclear organization), the PS externalization that appears immediately after nanoelectropulse exposure offers the possibility of characterizing within a relatively constrained and well understood system (the phospholipid bilayer membrane and the aqueous cytoplasmic and extracellular environment at the membrane boundary) the mechanism or mechanisms that directly mediate the interactions of pulsed electric fields with living cells.

We have now established that nanoelectropulse-induced membrane perturbation is field-driven and polarized (phospholipid scrambling occurs only at the anode-facing pole of the cell; the magnitude of the response follows the field strength), that shorter pulses require higher fields for lipid bilayer rearrangement (as would be expected for a mechanism that requires an increase in the membrane potential above the resting level), that calcium is not required (indicating that the calcium-sensitive enzymatic activities that maintain or scramble the normal membrane phospholipid asymmetry are not needed for the membrane reorganization that immediately follows pulse exposure), and that permeabilization of the plasma membrane to small molecules can be detected after nanoelectropulse exposure, albeit with higher fields and faster pulse repetition rates than are needed for visualization of membrane perturbation and intracellular calcium release. In addition, although localized FM1-43 fluorescence intensification cannot be taken in isolation as a specific indicator of PS externalization, previous studies of nanoelectropulse-driven PS translocation indicated by annexin V binding [7,12], and recent work combining annexin V and FM1-43 fluorescence imaging results and molecular dynamics simulations [39], point to a close association between the nanoelectropulse-induced membrane perturbations reported here and the PS translocation associated with ultra-short, high-field electric pulse exposure.

Current models of electroporation incorporate the well-established observation that the conductivity of a lipid bilayer increases by orders of magnitude when the transmembrane potential approaches 1 V, or even as low as 200 mV under some conditions [46]. Transient, aqueous, stochastic pore models [47,48] accommodate higher external electric fields with conductive pores that increase in number and diameter, clamping the membrane voltage at the critical, porating value [49]. Without this pore-dependent voltage regulation the transmembrane potential during a megavolt-per-meter pulsed field that lasts for only a few nanoseconds could rise to several volts, corresponding to a gigavolt-per-meter averaged across the 5 nm thickness of the lipid bilayer. Recent theoretical work [36,37] and experimental evidence [50] suggests that the membrane potential can increase to 1.5 V or more under these conditions, but determining what is really occurring on the nano scale – spatial and temporal – remains a challenge for those who model and those who measure.

Our observations of immediate phospholipid scrambling after exposure of cells to pulsed fields from 3 ns to 30 ns and 2 MV/m to 8 MV/m are consistent with the hypothesis that under the conditions of our experiments the critical membrane voltage is reached and exceeded, at least for a few nanoseconds, generating nanometer-diameter, nanosecond-duration openings in the lipid bilayer [48,51]. Although these pores would be expected to close quickly after the trailing edge of the pulse under physiological conditions [52], they would provide a low-energy path for migration of PS from the inner leaflet of the membrane to the outer surface. Repeated cycles of pulse-induced nanoporation would successively permit additional molecules of inner-membrane PS to find these tunnels to the external face of the cell.

**Figure 9:**
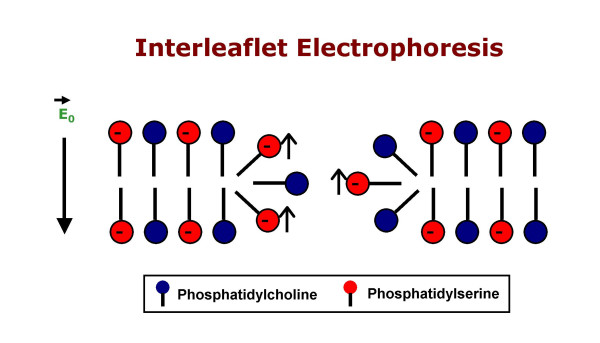
**Interleaflet electrophoresis**. Anionic phospholipids are electrostatically driven across the bilayer boundary at the periphery of a pore in the direction of the anode. This results in PS "flopping" (externalization) at the anode and "flipping" at the cathode (any PS present on the outer face of the membrane in the vicinity of a pore will be driven inward to the cytoplasmic leaflet of the bilayer).

Hydrophilic openings in the membrane, whether they originate stochastically or after the application of an external electric field (increasing the transmembrane potential lowers the energy barrier for pore formation, and in this sense electropores are stochastic), will facilitate translocation of inner layer phospholipids [17,47], but the strongly unipolar nature of nanoelectropulse-induced phospholipid perturbation and PS externalization suggests that the electric field contributes not only by driving the appearance of a sufficient number of membrane pores, but also by electrophoresing the PS head group, with its net negative charge, along the hydrophilic walls of the pore (Fig. 9). This interleaflet electrophoresis model is consistent with the observation of FM1-43 fluorescence intensification exclusively at the anode facing pole of the cell (and small but measurable reductions in FM1-43 fluorescence at the cathode face of the cell) even though membrane hyperpolarization and electroporation occur at both the anode and cathode poles.

Although this descriptive model may be sufficient to explain experimental observations at a broad level, it is predictive only in a qualitative sense. It should be considered progress nevertheless, since even a simplified engineering analysis presents substantial computational challenges, and the dynamic complexity of living biological structures raises a caution flag over even the most rigorous reduction of the cell to compartments of conductors and insulators. Future investigations will benefit from an ongoing synthesis, of hypothesis-driven data generation and data-constrained model hypotheses.

Most of the details that must be managed are susceptible to biophysical analysis. Pulse electrical properties (rise and fall time, shape), pore formation kinetics, distribution and migration of intracellular and extracellular ions after pore formation [53], electrical requirements and time course of PS electrophoresis through pores, the unknown but potentially significant role of membrane proteins in pore formation [54-57], and other complex parameters are all factors that may be incorporated at some point into molecular dynamics simulations or distributed network, transport lattice, or more abstract models. Molecular dynamics seems particularly suitable as a partner for experimental studies of the immediate interactions between the pulsed electric field and the cell. Initial work along these lines in our laboratory [39] and others [37,58,59], including simulations of PS externalization [39,60] indicates the utility of this approach.

In addition, we have presented evidence here for nanoelectropulse-induced small molecule permeabilization. Our previous inability to demonstrate membrane permeability after nanoelectropulse exposure, despite repeated attempts with many indicators and assays, represents a lack of sensitivity in the test methods and not an absence of the poration that theory, if not intuition, calls for. We can detect evidence of poration with our fluorescence microscopy system only if we use higher fields than have previously been available to us, and if they are delivered at a higher rate than we have used in our prior work.

It is not clear at this point whether the nanoelectropulse-induced YO-PRO-1 influx we observe is the result of the formation of hydrophilic pores in a process similar to long-pulse electroporation, or whether it is the consequence of activating purinergic receptor channels, perhaps specifically the P2X_7 _receptor channel, or some combination of these. Activation of the P2X_7 _receptor is associated with apoptosis [61], and YO-PRO-1 influx is used not only to detect P2X_7 _receptor activation [44,62] but also as a sensitive indicator of early apoptosis [43] which has been incorporated into a commercially available assay. This may be a key to the mechanism for nanoelectropulse-induced apoptosis. In our experiments we see a polarized entry of YO-PRO-1 into cells at both the anodic and cathodic faces, implying electroporation, but nanoelectropulse-induced activation of the P2X_7 _receptor channel or another member of the P2X receptor channel family cannot be ruled out on this evidence alone. It will also be of interest to investigate why higher pulse repetition rates increase the amount of YO-PRO-1 (and propidium iodide) that enters the cell. Experimentally verifiable schemes involving channel or pore opening set-up times and-or hysteresis in the channel or pore relaxation (closing) process that occurs when the pulsed field is withdrawn are not difficult to devise.

The localized electrically driven perturbation of the lipid bilayer that results in PS externalization serves as an experimental entry point to several paths of investigation. The event itself is biophysically interesting, and the individual components are well characterized, which makes it a reasonable focus for testing and refining models of the cell membrane, including more and more realistic membranes containing proteins, a heterogeneous population of phospholipids, and other lipid, protein, and polysaccharide species. Nanoelectropulse perturbation of cells and tissues may be expected to lead also to fruitful biochemical and cell biological investigations. The role of PS externalization in mediating cell clearance in immune and apoptotic processes is well known, and new signaling functions for PS externalization are coming to light [10], but it can also reflect regulatory modifications to cytoskeletal-membrane associations [24,25], and it may be possible to manipulate these cellular structures with nanoelectropulse treatment. Finally, the ability to nondestructively mark cells with a physiologically significant semaphore by means of a remotely delivered stimulus which could be targeted to specific cells and even specific regions of specific cells with a combination of varying pulse train recipes (amplitude, duration, pattern) and appropriate electrode placement and cell-binding ligands could be used to modulate cell activity and communication, including potentially the ability to induce malignant and other diseased cells to signal for their own removal [63,64].

## Conclusion

Electric pulses as short as 3 ns produce polarized (anode-directed) membrane disturbances that can be imaged with fluorescence microscopy and which are associated with phospholipid rearrangements and the influx of small molecules from the medium into the cytoplasm. Cellular responses to these previously uninvestigated very short pulse widths are similar to those observed with longer duration pulses and are consistent with a nanopore-facilitated, electrophoretic mechanism for the PS externalization observed after exposure of cells to nanosecond, megavolt-per-meter electric fields.

## Methods

### Cell lines and culture conditions

Human Jurkat T lymphoblasts (ATCC TIB-152) were grown in RPMI 1640 medium (Irvine Scientific, Irvine, CA) containing 10% heat-inactivated fetal bovine serum (FBS; Gibco, Carlsbad, CA), 2 mM L-glutamine (Gibco, Carlsbad, CA), 50 units/mL penicillin (Gibco, Carlsbad, CA), and 50 μg/mL streptomycin (Gibco, Carlsbad, CA) at 37 C in a humidified, 5% carbon dioxide atmosphere.

### Pulse generators and pulse exposures

For microscopic observations, cells were placed in a rectangular channel 100 μm wide, 30 μm deep, and 12 mm long, with gold-plated electrode walls, microfabricated with photolithographic methods on a glass microscope slide [65]. A fast MOSFET MicroPulser [66] was mounted on the microscope stage for delivery of 30 ns pulses directly to the microchamber electrodes in ambient atmosphere at room temperature as previously described [14]. For bipolar pulse exposures, a standard USC MicroPulser was capacitively coupled to the load, so that for every trigger event a negative pulse followed by a positive pulse was delivered to the cells (a low-value resistor provides a current return path for the second pulse). A USC-designed and fabricated NanoPulser, with fast recovery diode switching, provided pulses with widths less than 10 ns [67].

### Fluorescence microscopy and microphotometry

Observations of live cells during pulse exposures were made with a Zeiss Axiovert 200 epifluorescence microscope. For visualization of PS translocation with FM1-43 (Molecular Probes, Eugene OR; λ_ex _= 480 nm, λ_em _= 535 nm), cells were washed twice, resuspended in growth medium containing 5–20 μM FM1-43, incubated for 20 minutes at 37°C, and observed without additional washing. EGTA was from Calbiochem (EMD Biosciences, San Diego, CA), BAPTA-AM, DiI, propidium iodide (PI), and YO-PRO-1 were from Molecular Probes (Invitrogen, Eugene, OR), and Brilliant Blue G was from Sigma-Aldrich (St. Louis, MO). Images were captured and analyzed with a LaVision Imager QE camera and software (LaVision, Goettingen, Germany). Photometric data is from at least 20 representative cells from three independent experiments. Error bars represent the standard error of the sample mean.

## Authors' contributions

PTV designed and participated in the experiments and drafted the manuscript. YS contributed to the design of the experiments, conducted the fluorescence microscopy and imaging analysis, and participated in the preparation of the manuscript. MAG participated in the design and coordination of the study and in the preparation of the manuscript. All authors read and approved the final manuscript.
